# Dabigatran Induced Hemorrhagic Cystitis in a Patient with Painful Bladder Syndrome

**DOI:** 10.1155/2014/871481

**Published:** 2014-03-13

**Authors:** Helen Otteno, Erica Smith, R. Keith Huffaker

**Affiliations:** ^1^Department of Obstetrics and Gynecology, Quillen College of Medicine, East Tennessee State University, Johnson City, TN 37604, USA; ^2^Intermountain Budge OB/GYN Clinic, Logan Regional Hospital, Logan, UT 84341, USA

## Abstract

An 82-year-old female presented with longstanding history of both painful bladder syndrome and atrial fibrillation. She underwent hydrodistension remarkable for hematuria without temporary discontinuation of Dabigatran. Subsequently, patient was admitted to the hospital secondary to anemia and hemorrhagic cystitis.

## 1. Introduction

Dabigatran (Pradaxa) is a direct thrombin inhibitor which is used as an anticoagulant [1]. The recommended monitoring parameters for Dabigatran are less stringent than those for warfarin [2, 3]. Patients undergoing minor surgical procedures at minimal risk of bleeding may remain on Dabigatran. Dabigatran has not been previously associated with hemorrhagic cystitis.

## 2. Case

An 82-year-old female with painful bladder syndrome presented with worsening symptoms of pelvic pain, dysuria, frequency, and urgency. Her past medical history was significant for atrial fibrillation, cerebrovascular accident, arthritis, and hypothyroidism. Patient's past history was significant for a prior hydrodistension resulting in resolution of her symptoms for a few months. She underwent a CT abdomen and pelvis with/without contrast which revealed a diffuse thickening of the urinary bladder wall and no focal renal lesion or hydronephrosis.

After an in-office cystourethroscopy, which was significant for hypervascularity and decreased capacity, she underwent four bladder instillations, with a mixture of marcaine, heparin, and saline, without relief of her symptoms. After each instillation, patient complained of small volume gross hematuria. Hydrodistension of her bladder was scheduled. However, at the time of her scheduled hydrodistension, the bladder was noted to be filled with a large blood clot. Bladder washings and biopsy were obtained at this time and the hydrodistension was not completed. No focus of bleeding could be identified. Visualization was markedly diminished. Clinical followup was planned for 2 days later. The pathology from the washings and biopsy revealed bladder mucosa with chronic inflammation, negative for malignancy.

The patient subsequently was admitted to the hospital secondary to acute anemia, with a hematocrit (Hct) of 19.1% and a hemoglobin (Hgb) of 6.7 g/dL. She underwent transfusion with 4 units of PRBC, 5 units of frozen plasma, and 1 unit of platelets. An intravenous pyelogram revealed an intact bladder. Continuous bladder irrigation was performed.

Significant laboratory results included PTT-SSH of 26.3 (11.1–13.5) seconds, PT of 2.3 (9.7–12.9) seconds, and PTT of 123.0 (24–34) seconds. Dabigatran was discontinued and warfarin therapy was started. On day 2 of hospitalization, her Hct and Hgb increased to 25.5% and 9.0 g/dL, respectively. On hospital day 4, PT was 12 seconds, PTT 38 seconds, and Hct 29.5%. The patient was noted to have resolution of gross hematuria and she was discharged from the hospital without a catheter.

The need for anticoagulation secondary to atrial fibrillation was assessed prior to discharge, per internal medicine. Patient decided not to restart Dabigatran.

## 3. Discussion

One proposed mechanism for painful bladder syndrome is disruption of the glycosaminoglycan (GAG) layer which coats the inner surface of the bladder overlying the transitional epithelium. Deep into the transitional layer is the submucosa which contains microvasculature and overlies the detrusor muscle. Disruption of the GAG layer may result in increased exposure of the submucosa's vasculature. This exposure results in the appearance of hypervascularity with small hemorrhages in the bladder mucosa when performing a bladder hydrodistension [4].

With the proposed mechanism for the pathology of painful bladder syndrome resulting in exposure of the microvasculature of the bladder's submucosa, one can hypothesize that this layer is at increased risk of bleeding. Dabigatran results in an increased risk of bleeding without a method to easily reverse the bleeding [1]. The possibility of increased risk of hemorrhage from the bladder in a patient with painful bladder syndrome should be considered. Dabigatran has not been previously linked with hemorrhagic cystitis.

Given the possibility of significant bleeding, the asserted decreased risk of bleeding associated with Dabigatran may need to be considered further in patients undergoing minor procedures such as cystourethroscopy with or without hydrodistension. Even less invasive treatment modalities, such as bladder instillation therapies, may place these patients at increased risk for bleeding when taking Dabigatran.
